# Nutrient gaps and affordability of complementary foods in Eastern and Southern Africa and South Asia

**DOI:** 10.1093/nutrit/nuaa149

**Published:** 2021-03-08

**Authors:** Saul S Morris, Aashima Garg, Robert E Black

**Affiliations:** 1 Global Alliance for Improved Nutrition, London, USA; 2 UNICEF Headquarters, New York, New York, USA; 3 John Hopkins Bloomberg School of Public Health, Baltimore, Maryland, USA

**Keywords:** affordability, complementary feeding, nutrient gap, South Asia, Africa

Every parent aspires to provide a nutritious diet for their children, but many struggle to realize this ambition. The authoring agencies of the *2020 State of Food Security and Nutrition in the World* report^1^ estimated that one-quarter of the world’s population of > 1.5 billion people cannot afford even the cheapest possible nutrient-adequate diet that meets, according to the report, “all known requirements for essential nutrients.” According to the report, this diet is unaffordable for 53% of the population of sub-Saharan Africa, and 18% of the population of southern Asia, regions that, together, are home to 52% of the world’s children^2^ and a concentration of 86% of people living in extreme poverty worldwide.^3^

Young children, after 6 months of exclusive breastfeeding, require age-appropriate nutrient-dense foods in addition to breastmilk from 6 to 24 months of age to meet the needs of their rapidly growing bodies and brains.^4^ Well-established guidelines^5^ for this age group suggest that “meat, poultry, fish or eggs should be eaten daily,”^5^ as should vitamin A–rich fruits and vegetables. Providing such a diet is clearly impossible for the 185 million people (almost all in sub-Saharan Africa) who cannot afford even a single, cheap starchy staple in adequate quantities.^1^ These families do not have even enough maize meal to stave off hunger, so adding animal-source foods, fruits, or vegetables, even in small quantities, is a lot to ask. But for the remainder of the 1.5 billion people who can meet their basic energy needs yet still cannot afford all the nutrients their families should consume, it is conceivable that they could make improvements in the quality of their children’s diets by buying or growing just enough of the cheapest, locally available, nutritious foods. Children’s stomachs, after all, are much smaller than those of adults, so the quantities required to meet their needs are relatively small. This has been the underlying logic of decades of nutrition programming, which has attempted to convince parents to prioritize the needs of their very young children.

The unaffordability of nutritious foods, coupled with affordable non-nutritious foods, is a critical driver of poor quality of children’s diets, contributing to all forms of malnutrition. Headey and Alderman^6^ have shown that the food groups most likely to contribute to preventing undernutrition are precisely the ones that are most expensive to acquire (with cost expressed on a per-kilocalorie basis), and the cost differential is greatest in the regions of the world where child undernutrition is most prevalent. Even home production of such foods has a significant opportunity cost in terms of land, inputs, and family time. Therefore, it is critical to take recommendations to a greater degree of granularity: Exactly which locally available, nutrient-dense foods, in each context, are best matched to the specific nutritional needs of young children and are most affordable for the greatest number of families? This is the question addressed by the 5 research papers in this *Nutrition Reviews* supplement.

Answering this question is not straightforward. In an ideal world, we would know the current extent of multiple nutritional deficiencies in children, measured with a high level of accuracy, timeliness, and geographic disaggregation. We would also know the affordability of a wide range of local foods and their nutrient content in small volumes suitable for children’s consumption. Neither of these conditions is met in the real world, where we struggle with conflicting, dated, unrepresentative, and potentially biased information from multiple sources, and have no commonly agreed-on approach to describe and characterize the affordability of individual foods.

The first research article in the series^7^ introduces a novel methodology for identifying the public health significance of nutrient gaps in children’s diets. Eleven nutrients, each associated with child survival, growth, and development, are included. The methodology, called Comprehensive Nutrition Gap Analysis (CONGA), collates all relevant data points (ie, biomarker, functional, based on intake data of different kinds or supply-level data) from a given region and provides clear criteria for rating the nutrient gap (as negligible, low, moderate, or high) implied by each data point. This in itself is a major methodological contribution, because only a few, well-characterized biomarkers currently have consensus cutoff values that represent different levels of public health burden. The methodology then assigns weights to each data point on the basis of evidence type, geographic representation, recency of data collection, age and sex representation, and sample size. For each nutrient, an overall nutrient gap rating is derived (aggregating individual data points using the weight scores), as well as an evidence quality rating.

In the second research article in the series,^8^ White et al report detailed results from the application of this methodology to data from 6 countries in Eastern and Southern Africa. They report how many relevant data points could be identified in each country, and how many countries had nationally representative data (by nutrient and evidence type). The authors examine closely the quality of the available evidence and map the extent of deficiencies in the 11 nutrients, with each nutrient-country combination characterized by the certainty of the evidence located. The authors also discuss how many nutrient- and country-specific estimates had to be adjusted after discussion with local experts.

Beal et al, in the third research article in the series,^9^ report a comparable set of results from application of the CONGA methodology in all 8 countries of South Asia. In this article, there are clear differences in the availability of relevant data by country. In this report and that of White et al^8^ on the application of the CONGA methodology, the authors conclude by identifying a short list of regionally available foods that are particularly rich in the micronutrients identified as most likely to be missing in the diets of children living in the region.

The last 2 research articles in the series return to the 6 selected countries of Eastern and Southern Africa^10^ and the 3 largest countries in South Asia: Pakistan, India, and Bangladesh,^11^ mining recent household survey data to identify the most affordable foods that could close the deficits in consumption of key nutrients highlighted in the White et al^8^ and Beal et al articles.^9^ The authors first try to establish what purchased quantity of each candidate food would provide an amount of each prioritized micronutrient (or protein, or dietary energy) significant enough to contribute to reducing the observed nutrient gaps. They then cost such a portion using spatially and temporally matched price data and benchmark that cost against another set of data on the spending power of local households of different wealth profiles. Thus, the authors are able to draw conclusions about the foods that meet specific affordability thresholds for poorer and richer households in each country and are able to address critical nutrient intake problems for children both individually (nutrient by nutrient) and as a set. Findings are also presented disaggregated by place of residence (rural vs urban).

Together, these papers provide a strong basis for the design of interventions that could improve the nutrition of infants and young children in the world’s poorest regions,^2^ if implemented alongside other important improvements to services, behaviors, and the enabling environment. If the authors’ conclusions are valid, they can be taken as identifying a small set of foods that should be energetically promoted for the consumption of young children, knowing that they are locally available, affordable to the poorest households, and highly likely to contribute to reducing deficits in nutrients that are currently significantly under-consumed.

It is too early to say for sure whether these deductions are indeed valid. The authors make many assumptions about, among other things, the weights to assign to different types of evidence, the amount of a given nutrient that should reasonably be provided by a single food, and how much of the family budget could be spent on a single food intended exclusively for a subset of the household membership. These assumptions cannot be proven empirically and their predictive validity—whether promoting the foods identified would lead to improvements in the diets and nutritional status of children—will have to await the conduct of experimental studies. We can confidently assert that the CONGA methodology does appear to exhibit content validity because, by definition, it includes all different sources of information on each prioritized nutrient. However, the analyses also show that these information sources frequently do not agree, so the final rating is likely to be sensitive to the relative weightings applied to each type of evidence. The same analyses also show that careful review by local experts can lead to revisions of the ratings proposed by highly trained external experts, suggesting that published data on nutrient gaps can be hard to interpret, particularly if they are based on potentially conflicting data points.

Despite these uncertainties, the series provides an important step forward in evidence-based nutrition policymaking for infants and young children in poorer countries. The authors make transparent proposals for the structured triangulation of multiple data sources to understand priority nutrient gaps and set out a clear definition of affordability that includes unambiguous thresholds for the major data inputs. The researchers have shown that the method can be applied in a wide variety of settings, requiring no more than a few months of expert staff time, and that the findings are accessible to a broad set of policymakers in and beyond the studied regions. Building on this practical and transparent description of the methods, researchers will be able to extend findings to other settings, challenge key assumptions, and suggest refinements that may ultimately prove to be more valid.

## CONCLUSION

In the field of infant and young child nutrition, the challenge of affordability has not been given the emphasis it deserves. Only with the development of the Innocenti Framework on Food Systems for Children and Adolescents^12^ has the personal food environment (which includes affordability as a key domain) been given prominence. Efforts to improve infant and young child nutrition that promote unaffordable foods, or foods that do not provide key nutrients in adequate quantities, are doomed to failure. The world’s children deserve better.
